# Promoting and Optimizing the Use of 3D-Printed Objects in Spontaneous Recognition Memory Tasks in Rodents: A Method for Improving Rigor and Reproducibility

**DOI:** 10.1523/ENEURO.0319-21.2021

**Published:** 2021-09-29

**Authors:** Mehreen Inayat, Arely Cruz-Sanchez, Hayley H. A. Thorpe, Jude A. Frie, Blake A Richards, Jibran Y. Khokhar, Maithe Arruda-Carvalho

**Affiliations:** 1 Department of Psychology, University of Toronto Scarborough, Toronto, Ontario M1C1A4, Canada; 2 Department of Cell and Systems Biology, University of Toronto Scarborough, Toronto, Ontario M1C1A4, Canada; 3 Department of Biomedical Sciences, Ontario Veterinary College, University of Guelph, Guelph, Ontario N1G2W1, Canada; 4 Mila, Montréal, Quebec, H2S 3H1, Canada; 5 Department of Neurology and Neurosurgery, McGill University, Montréal, Quebec, H3A 2B4, Canada; 6 School of Computer Science, McGill University, Montréal, Quebec, H3A 2A7, Canada; 7 Learning in Machines and Brains Program, Canadian Institute for Advanced Research, Toronto, Ontario, M5G 1M1, Canada

**Keywords:** 3D printing, object recognition, open-source, rodents

## Abstract

Spontaneous recognition memory tasks are widely used to assess cognitive function in rodents and have become commonplace in the characterization of rodent models of neurodegenerative, neuropsychiatric and neurodevelopmental disorders. Leveraging an animal’s innate preference for novelty, these tasks use object exploration to capture the what, where and when components of recognition memory. Choosing and optimizing objects is a key feature when designing recognition memory tasks. Although the range of objects used in these tasks varies extensively across studies, object features can bias exploration, influence task difficulty and alter brain circuit recruitment. Here, we discuss the advantages of using 3D-printed objects in rodent spontaneous recognition memory tasks. We provide strategies for optimizing their design and usage, and offer a repository of tested, open-source designs for use with commonly used rodent species. The easy accessibility, low-cost, renewability and flexibility of 3D-printed open-source designs make this approach an important step toward improving rigor and reproducibility in rodent spontaneous recognition memory tasks.

## Significance Statement

Spontaneous recognition memory tasks are becoming standard in neuroscience labs studying cognitive function and using preclinical models of neurodegenerative, neuropsychiatric and neurodevelopmental disorders. However, variability in object selection across labs may hinder cross-lab comparisons and consensus across the field. Here, we discuss the advantages of, and optimization strategies for, the use of 3D-printed objects in rodent spontaneous recognition memory tasks, with the goal of increasing accessibility, reproducibility and rigor when running these tasks. We also share tested, open-source object designs for rats and mice with the broader scientific community.

## Introduction

Spontaneous recognition memory tasks have become a commonly used tool to assess cognitive function in rodents. These tasks rely on innate novelty preference in rodents to assess recognition memory for object features (novel object recognition; Mathiasen and DiCamillo, 2010; Leger et al., 2013; Lueptow, 2017), spatial location (object location; Vogel-Ciernia and Wood, 2014), recency of events (temporal order recognition; Barker et al., 2007; Barker and Warburton, 2011; Cruz-Sanchez et al., 2020), object category (object category recognition; Creighton et al., 2019), or a combination of features (e.g., object-place, object-in context, object-place-context, multisensory object oddity, object temporal location; Dix and Aggleton, 1999; Ameen-Ali et al., 2015; Cloke et al., 2016; Ramsaran et al., 2016a,b; Barker et al., 2017, 2020). The ability to recognize novel stimuli is critical for survival, emphasizing the ethological relevance of spontaneous recognition memory tasks. Furthermore, the simplicity of these tasks, which part with a need for prior training, food/water restrictions, specialized equipment or stress, makes them extremely accessible and versatile with considerable translational value (Winters et al., 2008; Raber, 2015). As a result, spontaneous object recognition tasks are increasingly being used as cognitive tests in rodent models of neurodegenerative, neuropsychiatric and neurodevelopmental disorders (Lyon et al., 2012; Grayson et al., 2015; Kent et al., 2018; Chini et al., 2020), to improve our understanding of the neural circuit basis of behavior (Winters et al., 2008; Warburton and Brown, 2015), as well as in studies assessing the impact of drugs and toxins on the brain (Dere et al., 2007; Goulart et al., 2010; Nanfaro et al., 2010; Schindler et al., 2010; Hamidullah et al., 2021). Indeed, a PubMed search for “object recognition in rodents” shows a 6-fold increase in publications in the past 15 years, with over 500 publications per year, capturing the continued growth in the popularity of these tasks.

While these advantages have made spontaneous recognition memory tasks a common feature of the behavioral neuroscience repertoire, multiple aspects of the tasks vary considerably between labs, which may hamper consensus and replication in the field. A major source of this variability stems from object selection (Spry et al., 2021). Objects used in spontaneous recognition memory tasks vary from small plastic toys/parts (Lee et al., 2005; Assini et al., 2009; Heyser and Chemero, 2012; Bath et al., 2017), empty glass or plastic bottles (Eacott and Norman, 2004; Heyser and Chemero, 2012), jars/bottles filled with colored rocks or sand (Sik et al., 2003; Reger et al., 2009; Cyrenne and Brown, 2011; Leger et al., 2013), soda cans (Ramsaran et al., 2016b), candle sticks (Eacott and Norman, 2004; Annie Vogel-Cierna, 2015) to LEGO/Mega Bloks figures (Hannesson et al., 2004; Norman and Eacott, 2004; Krüger and Hanganu-opatz, 2013; Leger et al., 2013; Lins et al., 2014; Barker and Warburton, 2015; Travaglia et al., 2018). This variability in the objects used introduces additional confounds in the experimental process. Ideally, spontaneous recognition memory tasks would use objects that can be shared across labs.

Luckily, the increased availability and low cost of 3D printing has made it possible for 3D-printed parts to be integrated into a variety of behavioral neuroscience experiments and tools. In our experience, 3D-printed objects using polylactic acid (PLA) or polyethylene terephthalate glycol (PETG) filaments cost approximately USD$1–3 per printed object depending on object height and fill density used. This has opened the door to many open source tools and apparatuses in the field, such as automated feeding assessment devices (Matikainen-Ankney et al., 2021), automated drinking and lick incidence measurement apparatuses (Frie and Khokhar, 2019; Godynyuk et al., 2019), automated catalepsy measurement tools (Luciani et al., 2020), and capacitance sensor based objects for object recognition (Spry et al., 2021). In the case of spontaneous recognition memory tasks, the use of 3D-printed objects offers the additional advantages of increased design flexibility, uniformity across labs and renewability, thereby increasing rigor, reproducibility and consistency over time. Lastly, consistency in the objects used across institutions could facilitate the creation of large-scale object recognition datasets that could be used to generate hypotheses and test novel questions, similar to what has been done for touch-screen based testing (Beraldo et al., 2019). Here, we provide a perspective on how best to promote and optimize 3D-printed objects for spontaneous recognition memory tests in rodents.

## Challenges Associated with Object Selection in Spontaneous Object Recognition Tasks

One of the major challenges for rodent object recognition tasks is that specific object features inherently affect exploration (Heyser and Chemero, 2012). This is because rodents display innate biases for certain object features, and some object dimensions may induce threatening or anxiogenic elements to the task, particularly in younger rodents (Reger et al., 2009; Cruz-Sanchez et al., 2021). Moreover, the level of similarity between the objects, or feature ambiguity, influences task difficulty and underlying brain circuit recruitment (Norman and Eacott, 2004).

While studies advocate for uniformity in the size and complexity of objects (Silvers et al., 2007), this becomes a challenge when using ready-made/store-bought objects. A considerable proportion of spontaneous recognition memory studies do not provide details on the objects used or the baseline preference for these objects, an obstacle for cross-lab comparisons, as well as assertions on feature ambiguity between sampled objects. While the use of LEGO/Mega Bloks figures overcomes some of those barriers, and affords considerable design flexibility, those constructs are still limited in design options and surface area uniformity when compared with 3D-printed objects. Lastly, while touch-screen based objection recognition tasks have also gained popularity (Romberg et al., 2013), and provide valuable translational consistency with tasks used in humans and various options for object optimization, their cost can be prohibitive.

Another issue that presents itself is the additional variability that is introduced when these tasks are used with animal models of neurologic or psychiatric disease, where an animal might display dysfunctions in perception, locomotion, or display anxiety-like behaviors that may cause the animal to stay close to the edges of the arena. These phenotypes may, therefore, impact the animal’s ability to perceive or interact with the object features, especially if the objects are not uniform in their appearance. As an example, an animal sticking close to the edge of an open field arena may only interact with the back of an object that has varying features on all its sides, thereby affecting the ability of the animal to assess object novelty, or inducing changes in baseline preference for the object.

## The Benefits of Using 3D-Printed Objects

**Figure 1.**
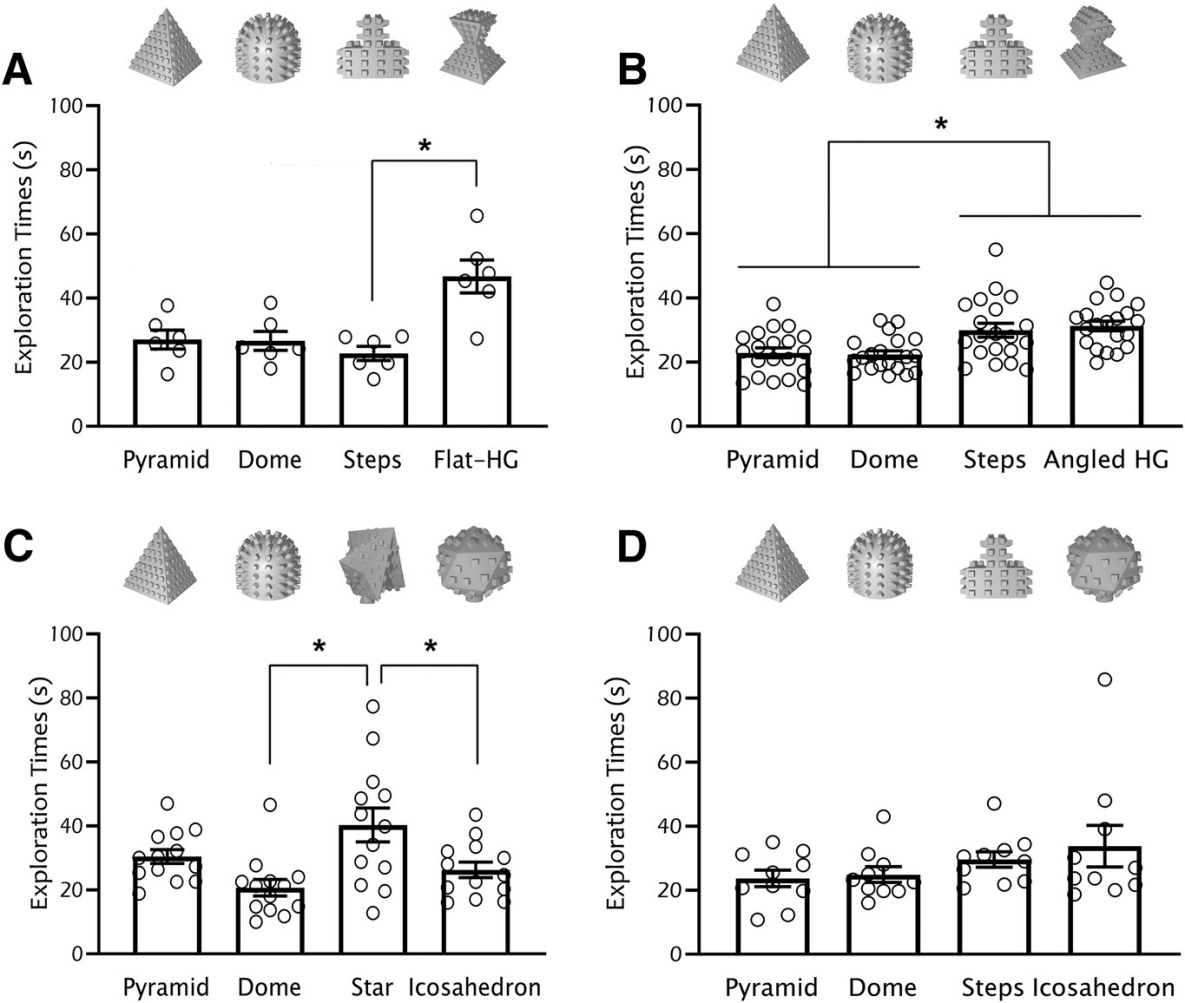
Example data for optimizing the choice of four objects in C57/129J mice. Starting from two previously tested objects (dome and steps), we proceeded to add two new objects with the goal of finding a non-biased four-object combination. When finding innate preference for one or more objects (***A***), possible strategies are to (1) modify features that drive bias (***B***; for example, by removing the flat top in the flat hourglass design); or (2) replace preferred objects until finding an ideal object combination (***C***; for example, we introduced two novel designs, star and icosahedron, but found innate preference for the star object; this was then followed by ***D***, when we replaced the star with the steps object to finally see an absence of innate preference). Multiple rounds of tests may be necessary, but in our experience, innate preferences are quite robust across animal cohorts in future experiments. Bars represent mean exploration time ± SEM. HG, hourglass; *p < 0.05. Methodology: adult C57/129J (a cross between C57BLK/6J and 129S1/SvImJ strains) mice were handled and habituated (5 min of handling followed by placement into the behavioral chamber for 4 min) to the square open-field behavioral chamber (30 × 30 × 30 cm) twice a day for four consecutive days. On the following day, mice were placed back into the chamber to interact for 5 min with four objects located 3 cm away from each wall at each corner of the chamber. Exploration time (seconds) per object was measured using ANY-maze software. Object location and start position within the chamber were counterbalanced and randomized across subjects. All animal procedures were approved by the local Animal Care Committee. Statistics: (***A***) one-way RM ANOVA *F*_(3,15)_ = 7.53, *p* = 0.011; Tukey’s *post hoc* test shows a significant difference between steps and flat HG *p* = 0.04; (***B***) one-way RM ANOVA *F*_(3,57)_ = 9.10, *p* = 0.0003; Tukey’s *post hoc* test shows a significant difference between pyramid versus steps *p* = 0.042, pyramid versus angled HG *p* = 0.0004, dome versus steps *p* = 0.031, dome versus angled HG *p* = 0.0003; (***C***) one-way RM ANOVA *F*_(3,36)_ = 5.59, *p* = 0.003; Tukey’s *post hoc* test shows a significant difference between dome versus star *p* = 0.002, star versus icosahedron *p* = 0.036; (***D***) one-way RM ANOVA *F*_(3,27)_ = 1.51, *p* = 0.25.

In recent years, 3D printers have become cheaper and more easily accessible, turning them into a staple at research institutions across the world. Their ubiquity provides an opportunity to increase cross-lab consistency in object selection in spontaneous recognition memory tasks. The flexibility afforded by 3D-printed designs is multi-fold. 3D-printed designs minimize the number of variables driving exploration by matching texture, color, scent, dimensions and surface area across all objects. Changing the filament type can also allow for integration of multi-sensory factors [e.g., using thermoplastic polyurethane (TPU) to create softer objects compared with PLA or acrylonitrile butadiene styrene (ABS)]. Furthermore, objects can achieve any degree of symmetry or subtle manipulations of feature ambiguity, offering ample opportunity for discreet and iterative investigations of object processing. This flexibility is further critical when troubleshooting innate object bias, since any feature driving bias can easily be removed or modified into a new design, and new objects promptly printed for use (see an example in Fig. 1).

Importantly, 3D-printed objects also surmount the challenge of long-term object availability, which often affects continuity when using store-bought objects. Greater uptake of the use of open-source 3D-printed object designs would facilitate comparisons of studies across labs, improving reproducibility and increasing rigor in the interpretation of recognition memory data. Lastly, the relative affordability of these objects could help to improve the uptake of these methods in underfunded research environments.

## Strategies for Optimizing the Use of 3D-Printed Objects

### Always test for bias

As a valuable case study, when one of the authors first started using 3D-printed object designs for novel object recognition, they thought they had been careful because they used designs from another lab. They also made sure to counterbalance objects and sides in their experimental design. Unfortunately, when analyzing their data, they realized that animals showed increased preference when one of the objects was assigned as the novel object, because of innate preference for that object’s features, and the data had to be discarded. This cautionary tale emphasizes why excluding the possibility of innate object bias should be the first step when testing any new object combination, and an important analysis to include when publishing data using recognition memory tasks. Importantly, bias should always be assessed in well-handled and habituated animals, as that in itself is a major variable in determining exploration (Powell et al., 2004; Jablonski et al., 2013; Annie Vogel-Cierna, 2015; Cruz-Sanchez et al., 2021).

### Mice are not small rats, and neither species is homogenous

**Figure 2.**
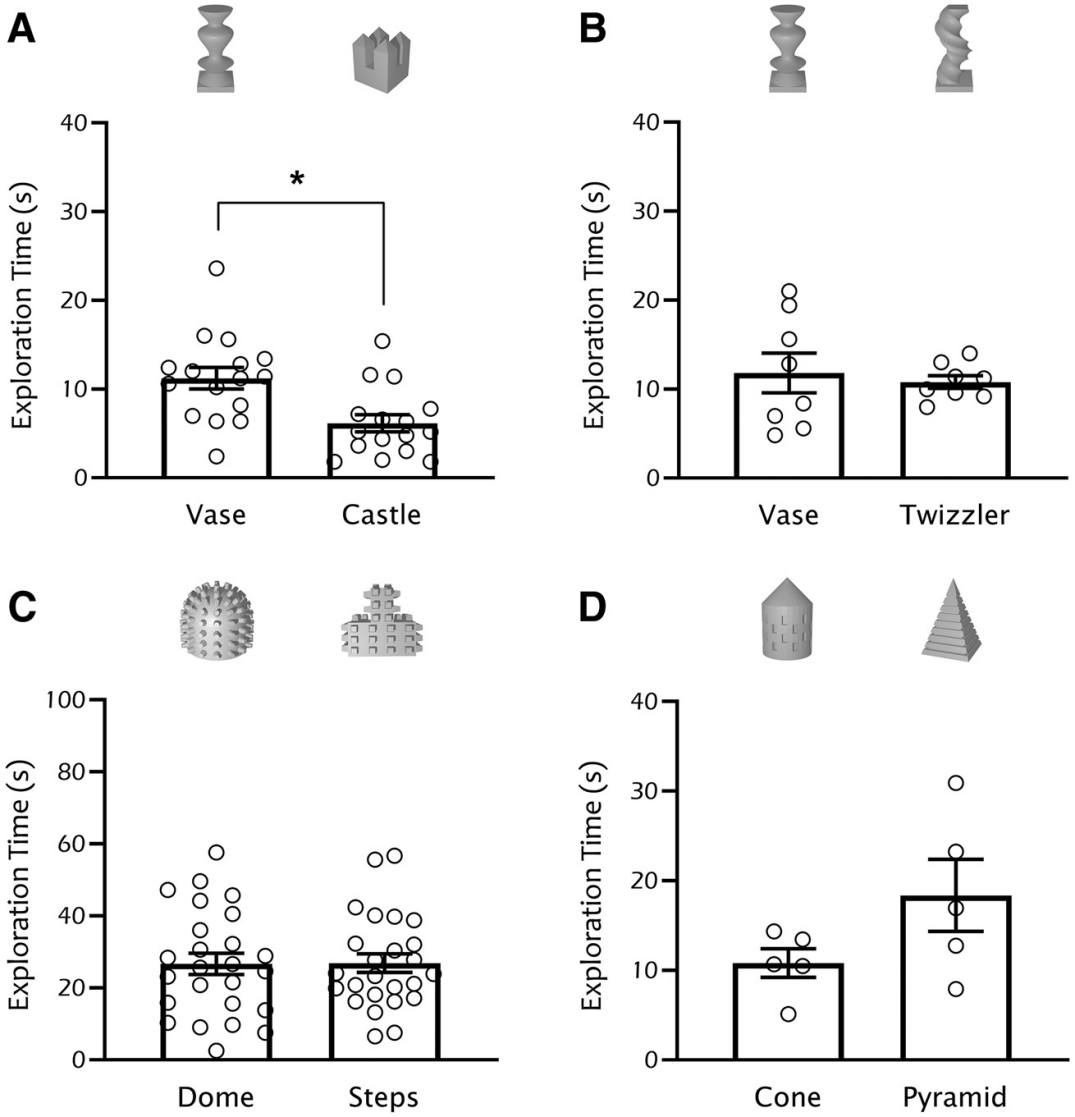
Example data for objects tested in rats, C57/129J and C57BL/6N mice. ***A*, B**. Sprague Dawley rats showed a preference for objects closer to their height (vase or twizzler) when compared with smaller objects (castle). ***C***, ***D***, Example of two-object combinations tested with C57/129J (***C***) and C57BL/6N (***D***) mice that showed similar innate preference. Bars represent mean exploration time ± SEM; *p < 0.05. Methodology: (***A***, ***B***) adult Sprague Dawley rats were handled for 2 min and habituated to a square open field (50 × 50 cm) for 5 min each day for the 3 d before experiments. The following day, rats were placed in the open field facing the wall away from the objects with different pairs of objects placed equidistant from each other and the wall (12.67 cm). Noldus EthoVision software was used to measure cumulative exploration time per object for 5 min with a threshold distance of 2 cm from the object indicating exploration. Left and right object location within the chamber was counterbalanced. All animal procedures were approved by the local Animal Care Committee. ***C***, Adult C57/129J mice were handled and habituated as described in Figure 1. On the day of testing, mice were placed into the chamber facing the wall located opposite the objects. Objects were placed in the northwest and northeast corners of the chamber, 3 cm away from each wall. Mice explored the two objects for 5 min. Exploration time (seconds) per object was measured using ANY-maze software. Object location within the chamber was counterbalanced. All animal procedures were approved by the local Animal Care Committee. ***D***, Adult C57BL/6N mice were handled for 2 min/d across three days before habituation and testing. To habituate mice to the behavioral chamber (45 × 45 × 30 cm), animals were allowed to freely explore the open field for 10 min/d across two consecutive days. On the following day, mice were placed back into the open field for 10 min with two unique objects in adjacent corners of the field. Objects were positioned 10 cm from each wall, and the corner in which each object was placed was counterbalanced across trials. Noldus EthoVision software was used to quantify object exploration with a distance threshold of 2 cm from the object indicating exploration. All animal procedures were approved by the local Animal Care Committee. Statistics: (***A***) paired *t* test *t*_(15)_ = 3.28, *p* = 0.006; (***B***) paired *t* test *t*_(7)_ = 0.49, *p* = 0.36; (***C***) paired *t* test *t*_(24)_ = 0.05, *p* = 0.95; (***D***) paired *t* test *t*_(4)_ = 2.39, *p* = 0.075.

Considerable species (Blaser and Heyser, 2015) and strain (Sik et al., 2003; Ennaceur et al., 2005; Kim et al., 2005; van Goethem et al., 2012) differences in exploration have been reported in spontaneous recognition memory tasks. As different species and strains vary in the way they approach novelty, it is very likely that object combinations that work for a specific species/strain might not translate to another, both in terms of exploration time and bias. As one example, two of the authors’ labs had tested and optimized the use of pegs for increasing exploration in C57/129J mice. However, when another author tried these tested designs in rats, they found that the rats chose to climb and bite the pegs instead, and in turn had to optimize completely new object designs (Fig. 2). If a lab is planning to conduct cross-species (or cross-strain) comparisons, testing object combinations should ideally be done in all relevant species/strains from the start, to avoid this scenario. Similarly, while the open-source designs and combinations offered here (Figs. 1, 2; https://www.thingiverse.com/thing:4964466 and https://www.thingiverse.com/thing:4964541) have worked well within the specified species/strains in tests of novel object recognition, object-location, temporal order recognition (Cruz-Sanchez et al., 2020) and object-place, they must be re-validated before large-scale experiments taking place, especially in novel strains or transgenic animals.

### Less is more

When minimizing bias across objects, a main lesson we have learned is that, counter-intuitively, objects with the highest preference are likely the best ones to avoid. We have spent a considerable amount of time designing objects to match the level of innate preference of a particularly preferred object, only to painfully realize it is easier to simply discard a highly preferred object and use two equally, but less preferred ones (Fig. 1). Provided that animals are still spending enough time exploring the objects, in our experience, excluding objects with high innate preference is a more efficient strategy for finding object combinations with equivalent innate preference, as long as any potential floor effects on object preference can be avoided.

### Features that can be modified to alter bias

Many strategies can be employed to optimize object selection and minimize innate bias. The difficulty of choosing object combinations with equivalent innate preference is directly proportional to the number of objects in the task. While optimizing object selection for two-object tasks such as novel object recognition or temporal order recognition may seem like a feasible challenge, this becomes complex for four-object tasks such as object-place (Fig. 1). Our labs have identified some of the object features that affect exploration. While by no means exhaustive, this list could aid others in employing strategies to optimize 3D-printed object selection in spontaneous recognition memory tasks:

#### Height of objects

The height of the object can affect exploration in different ways, relative to the size of the animal. While objects with heights above that of the animal may reduce exploration, likely by inducing anxiety-like behavior (this may particularly affect younger rodents; Reger et al., 2009), if objects are too short they might incite climbing/laying (Fig. 2*A*). A few developmental studies have proposed to scale the size of objects according to animal size (Ricceri et al., 2000; Reger et al., 2009; Travaglia et al., 2018), something easily achievable with 3D-printed designs. Our data with rats suggest this may also be applicable across species, with rats seemingly preferring taller objects (Fig. 2*A*). While, to the best of our knowledge, this scaling strategy has not been directly investigated, we expect object height would affect exploration rates across ages and species/strains if objects are too tall or too short. While we have not directly tested variations in object volume, we hypothesize the same principle would apply, and recommend selecting object dimensions that are similar to animal size.

#### Protrusions and indentations

Introduction of concave or convex elements to 3D-printed objects may affect exploration. We have found that C57/129J mice display a preference for protrusions (e.g., pegs), which increase object exploration in this strain. In contrast, rats climb on pegs instead of exploring the objects. We have found no increase in cleanup time when using pegged objects, provided objects are properly sprayed in between trials.

#### Flat tops

In our experience, C57/129J mice tend to lay considerably more on 3D-printed objects with a large flat top, when compared with objects with an angled or curved top. Anecdotally, the object with the most preference two of our labs have found for this strain was a cube with pegs, which substantially exceeded all other tested objects in preference, and was therefore never used. From this and other observations, we try and minimize using objects with large flat surfaces and square angles when working with this strain.

### Technical recommendations

When 3D printing objects for rodent object recognition tasks, we recommend using the same filament (we have tested PLA and PETG filaments) and color for all printed objects, to minimize variables. We have not directly tested whether changes in texture from filament type affect object exploration, but saw similar exploration levels between PLA and PETG filament printed objects. If using automated tracking software, choosing a filament color with the most contrast with the animal coat color will improve tracking. Our labs have used different strategies for immobilizing objects in the arena. Importantly, properly immobilizing objects will minimize any variability in exploration because of object weight, since all objects will be equally immovable by the animal. One option is to use an arena with a metallic floor and attach magnets to the 3D-printed object (Cruz-Sanchez et al., 2020). This allows flexibility in the location of the objects, while securing the objects in place throughout trials. Another useful alternative that can be used with a variety of surfaces and materials is 3M Dual Lock strips. These strips can be attached to the arena and objects, locking them in place. Importantly, Dual Lock strips are able to maintain adhesion to surfaces through cage washes, and the objects can be used interchangeably between various arenas. Finally, sticky tack can also be used to immobilize objects. Sticky tack allows for a smaller gap between the object and the floor when compared with the Dual Lock strips, is reusable and easily accessible. However, while sticky tack was strong enough to hold objects in place for tasks with C57BL/6N mice, it is not recommended for recognition tasks using rats.

## Conclusion

With the increasing popularity of spontaneous object recognition task variants to assess memory in rodents, approaches that help to standardize these tasks will help improve rigor and reproducibility. Here, we present a case for the use of 3D-printed object designs, and provide a framework for assessing the suitability of these objects across species and task variations. Moreover, through providing validated designs for 3D-printed objects, we hope to promote the harmonization of approaches and methods toward more generalizability of spontaneous object recognition findings.

## Synthesis

Reviewing Editor: Mark Laubach, American University Decisions are customarily a result of the Reviewing Editor and the peer reviewers coming together and discussing their recommendations until a consensus is reached. When revisions are invited, a fact-based synthesis statement explaining their decision and outlining what is needed to prepare a revision will be listed below. The following reviewer(s) agreed to reveal their identity: Michael Totty.

Two expert reviewers read your manuscript and provided the reviews given below. Essential revisions are given below each review based on discussion of the paper and reviews in the consultation session.

Reviewer #1

This well-written manuscript details the current lack of standardization of objects used in rodent spontaneous recognition memory tasks (i.e., novel object recognition). Recognition memory tasks leverage rodents’ innate preference for novelty to assess memory for familiar vs novel objects. However, specific object features can bias exploration time and confound results. The authors discuss advantages of using 3D printed objects to not only standardize objects used across labs, but also optimally select for objects with desired innate preference. They also provide an open-source repository for 3D printing objects as well as recommendations for objection selection and controlling for potential biases. Collectively, this should be a valuable resource for many neuroscientists.

As the authors noted, one of the strengths of recognition memory tasks is the low cost. Can the authors provide in the text a rough estimation for the cost of 3D printing these objects with the recommended filaments (PLA and PETG)?

General statistical information should be provided for the data shown in the figures (which test was used, p-values, error bars, etc.)

EDITORIAL FEEDBACK

Please be sure to address the two points raised in the review above in your revised manuscript.

Reviewer #2

The opinion manuscript entitled “Promoting and Optimizing the Use of 3D Printed Objects in Spontaneous Recognition Memory Tasks in Rodents: A Method for Improving Rigor and Reproducibility” provides a critical avenue to address the discrepancies that arise during object recognition test. Authors have explicitly tested the different objects to check their inherent preferences for the objects. The manuscript is well thought and nicely written; however, I have the following major issues.

1. While authors tried to scrutinize the different shapes of the object, they have not explicitly tested the other factors like texture, material types to be used or weight, to understand exactly which objects should be used and which should be avoided. This will be helpful for researcher who cannot have 3D printing facility.

2. Although authors have tested different objects shapes, its not clear whether these objects do work in the real experiments, as their similar texture would be difficult to recognize during novel object recognition. Authors should explain that the possible specifications for novel object in this test.

3. What could be the reasons for significant differences in the object exploration for first two vs last 2 object in Fig. 1B and C? Can authors please state the conclusion, clearly?

4. Authors should explain how difficult it would be to clean the objects between animal trials as they seem to have the dotted structure.

5. It’s not clear that how authors tested these objects, what were the habituation, or experimental light conditions.

6. Do authors think that these objects can also be used for object place recognition tests as the place recognition would depend on concurrently with the context along with the object preference?

7. Do authors think if overall volume of the object could also affect the exploration time?

EDITORIAL FEEDBACK

The seven issues above were discussed in the consultation forum and the following text from Reviewer #2 summarized the changes that should be made:

"For more clarification on my comments, since this is an Opinion article, I feel that authors can add discussions on texture, material types, and weight of the objects in the manuscript. Authors can also add their opinion on usefulness of the objects used in the manuscript, in real set-ups of object recognition tests. They can clarify few things mentioned in my comments and not to test them explicitly.”

## Author Response

We thank the reviewers and editor for their time, generous comments and excellent suggestions. We believe the revised manuscript is much improved as a result of this review process. We have addressed and incorporated all issues raised in the revised manuscript and include a detailed response to each point below:

Reviewer #1

As the authors noted, one of the strengths of recognition memory tasks is the low cost. Can the authors provide in the text a rough estimation for the cost of 3D printing these objects with the recommended filaments (PLA and PETG)?

Thank you for raising this important point. We have added a sentence including this cost. Specifically, whereas an average 1kg spool of PLA or PETG filament usually cost USD$20-25, in our hands that allows for printing of 20 or so objects for mice, and slightly fewer for rats, depending on the height of the object as well as fill density.

General statistical information should be provided for the data shown in the figures (which test was used, p-values, error bars, etc.)

We thank the reviewer for catching this oversight. We have incorporated statistics as well as a brief methodological description into the figure legends. In the process, we also noticed a small error on the representation of significance in Figure 1A and have corrected it in this version of the manuscript.

Reviewer #2

The opinion manuscript entitled “Promoting and Optimizing the Use of 3D Printed Objects in Spontaneous Recognition Memory Tasks in Rodents: A Method for Improving Rigor and Reproducibility” provides a critical avenue to address the discrepancies that arise during object recognition test. Authors have explicitly tested the different objects to check their inherent preferences for the objects. The manuscript is well thought and nicely written; however, I have the following major issues.

1. While authors tried to scrutinize the different shapes of the object, they have not explicitly tested the other factors like texture, material types to be used or weight, to understand exactly which objects should be used and which should be avoided. This will be helpful for researcher who cannot have 3D printing facility.

The reviewer is correct in that we have only tested PLA and PETG filaments, and cannot speak to differences in innate preference with other 3D printing filaments. In terms of weight, all objects are firmly immobilized on the chamber, and therefore we do not believe weight would affect exploration, given that animals are unable to tilt or displace the objects in any way. We have discussed this in the revised manuscript. Additionally, previous studies have usually only modified the shape of the objects, while keeping the materials consistent (e.g., legos or touch-screen).

2. Although authors have tested different objects shapes, its not clear whether these objects do work in the real experiments, as their similar texture would be difficult to recognize during novel object recognition. Authors should explain that the possible specifications for novel object in this test.

We thank the reviewer for noticing that we had failed to mention that these object designs have been successfully used in our labs for novel object recognition, object-location, temporal order recognition and object-place tests. We have incorporated this information in the revised manuscript, although we were unable to self-cite due to eNeuro double-blind review policy.

3. What could be the reasons for significant differences in the object exploration for first two vs last 2 object in Fig. 1B and C? Can authors please state the conclusion, clearly?

While it can be difficult to assess directly which object features drive innate preferences, and would require extensive experimentation, our attempt was to offer an example of sequential steps in optimizing object selection for a 4-object combination. We have raised a few features that in our experience increase innate preference (e.g. flat top surface), which may have contributed to the skewed preferences depicted in the figure, but we propose that perhaps a more optimal strategy is to exclude objects with higher innate preference and test other designs. We have re-written the figure legends to clarify this further.

4. Authors should explain how difficult it would be to clean the objects between animal trials as they seem to have the dotted structure.

Although seemingly challenging, by spraying the objects with 70% ethanol and drying them with a paper towel, in our experience cleaning of pegged objects does not increase cleanup up time significantly when compared to smooth objects. We have added a note about that to the revised manuscript.

5. It’s not clear that how authors tested these objects, what were the habituation, or experimental light conditions.

We thank the reviewer for bringing this up. We have added a detailed methodology section to the figure legends to incorporate this information.

6. Do authors think that these objects can also be used for object place recognition tests as the place recognition would depend on concurrently with the context along with the object preference?

Yes, and we have successfully used them in mice for object place tasks. This is now mentioned in the manuscript.

7. Do authors think if overall volume of the object could also affect the exploration time?

We have observed that object height affects exploration in rats, but have not tested whether overall volume does. We imagine the same principle should apply, and tried to keep dimensions closer to animal size. We have added a sentence addressing this possibility to the revised manuscript.

EDITORIAL FEEDBACK

The seven issues above were discussed in the consultation forum and the following text from Reviewer #2 summarized the changes that should be made:

"For more clarification on my comments, since this is an Opinion article, I feel that authors can add discussions on texture, material types, and weight of the objects in the manuscript. Authors can also add their opinion on usefulness of the objects used in the manuscript, in real set-ups of object recognition tests. They can clarify few things mentioned in my comments and not to test them explicitly.”

We appreciate this clarification and have added text discussing texture and material type, weight, as well as experimental use of the objects to the manuscript.
